# A roadmap to precision treatments for familial pulmonary fibrosis

**DOI:** 10.1016/j.ebiom.2024.105135

**Published:** 2024-05-07

**Authors:** Killian Hurley, Mari Ozaki, Quentin Philippot, Liam Galvin, David Crosby, Mary Kirwan, Deborah R. Gill, Konstantinos-Dionysios Alysandratos, Gisli Jenkins, Matthias Griese, Nadia Nathan, Raphael Borie, COST Open-ILD Group Management Committee, Killian Hurley, Deborah Snijders, Nicolaus Schwerk, Nico Lachmann, Matthias Griese, Daniel O'Toole, Raphael Borie

**Affiliations:** a Department of Medicine, Royal College of Surgeons in Ireland, Education and Research Centre, Beaumont Hospital, Dublin 9, Ireland; b Tissue Engineering Research Group, Royal College of Surgeons in Ireland, Dublin 2, Ireland; c Université Paris Cité, Inserm, PHERE, Hôpital Bichat, AP-HP, Service de Pneumologie A, Centre Constitutif du Centre de Référence des Maladies Pulmonaires Rares, FHU APOLLO, Paris, France; d Physiopathology and Epidemiology of Respiratory Diseases, Inserm U1152, UFR de Médecine, Université Paris Cité, 75018, Paris, France; e European Pulmonary Fibrosis Federation, Overijse, Belgium; f Irish Lung Fibrosis Association, Ireland; g Department of General Practice, Royal College of Surgeons in Ireland, Dublin, Ireland; h UK Respiratory Gene Therapy Consortium, London, United Kingdom; i Gene Medicine Research Group, Radcliffe Department of Medicine (NDCLS), University of Oxford, Oxford, United Kingdom; j Center for Regenerative Medicine, Boston University and Boston Medical Center, Boston, MA, 02118, USA; k Imperial College London, 4615, National Heart & Lung Institute, London, United Kingdom of Great Britain and Northern Ireland; l Department of Pediatric Pneumology, German Center for Lung Research (DZL), Dr von Hauner Children's Hospital, Ludwig-Maximilians-University, Munich, Germany; m Sorbonne Université, Pediatric Pulmonology and Reference Center for Rare Lung Diseases RespiRare, Inserm U933 Laboratory of Childhood Genetic Diseases, Armand Trousseau Hospital, APHP, Paris, France; n The Pulmonary Center and Department of Medicine, Boston University Chobanian & Avedisian School of Medicine, Boston, MA, 02118, USA

**Keywords:** Familial pulmonary fibrosis, Interstitial lung disease of genetic cause, precision medicine, Pulmonary fibrosis, Preclinical models, Telomere related gene, Surfactant related gene, Telomeropathy, Induced pluripotent stem cells

## Abstract

Interstitial lung diseases (ILDs) in adults and children (chILD) are a heterogeneous group of lung disorders leading to inflammation, abnormal tissue repair and scarring of the lung parenchyma often resulting in respiratory failure and death. Inherited factors directly cause, or contribute significantly to the risk of developing ILD, so called familial pulmonary fibrosis (FPF), and monogenic forms may have a poor prognosis and respond poorly to current treatments. Specific, variant-targeted or precision treatments are lacking. Clinical trials of repurposed drugs, anti-fibrotic medications and specific treatments are emerging but for many patients no interventions exist. We convened an expert working group to develop an overarching framework to address the existing research gaps in basic, translational, and clinical research and identified areas for future development of preclinical models, candidate medications and innovative clinical trials. In this Position Paper, we summarise working group discussions, recommendations, and unresolved questions concerning precision treatments for FPF.

## Introduction

Although several review articles and opinion pieces have been published on finding future treatments and cures for ILD, in particular for idiopathic pulmonary fibrosis (IPF), these do not specifically address FPF. Early clinical studies of novel and repurposed medications have been completed or are underway NCT04638517, NCT03710356.^1^^,^^2^ Data from emerging preclinical disease models suggests that we can recapitulate disease processes in monogenic FPF allowing us to further explore disease pathogenesis and test existing and emerging drug therapies. However, there are many gaps in our knowledge. Overall, a framework or roadmap is needed to address these gaps and to describe the steps which are needed to find and test new precision treatments. A working group of adult and children's ILD physicians, scientists, patients, legal and disease registry experts, and biobank experts was convened at the COST funded Open-ILD Training School in Dublin.

**Search strategy and selection criteria**

We searched PubMed and MEDLINE for papers published in English from database inception to January 31st, 2024, with multiple combinations of the terms “familial pulmonary fibrosis”, “genetic interstitial lung disease”, “pulmonary fibrosis”, “disease modelling”, “pathophysiology”, “clinical trials“, “diagnosis”, and “treatment”. Abstracts were reviewed to assess potential relevance for this paper and full papers were obtained for those selected based on novelty and clinical relevance. Conference abstracts were not considered. This search strategy was supplemented by the authors’ own literature searches for their meeting presentations and for workshop summaries at the COST funded Open-ILD Training School in Dublin in 2022. When publications with overlapping content were identified, the references deemed most immediately relevant were included in the final citation list.

**Key Messages**

• Genetic factors may cause or contribute significantly to the risk of developing ILD in children and adults, now described as familial pulmonary fibrosis (FPF).
• Patients with a monogenic cause of ILD may have a worse prognosis than sporadic ILD and respond poorly to current treatments.
• Robust preclinical models of FPF are lacking as there is limited access to tissue that faithfully recapitulates normal and abnormal human lung creating a major hurdle to developing new therapies.
• A growing literature suggests epithelial dysfunction as an initiating event in ILD.
• Lung cells derived from patient pluripotent stem cells (PSCs) are an emerging system to model FPF *in vitro.*
• Large registries and biobanks of well-phenotyped patients with FPF are central to facilitate the molecular and cellular origins of disease and to identify new treatments and cure.
• Prospective clinical trials of precision therapeutics such as telomere specific treatments for patients with telomere related gene (TRG) associated ILD are ongoing and early clinical trials of non-specific and anti-fibrotic medications for patients with surfactant related gene (SRG) associated ILD have been attempted.
• More specific treatments such as gene therapies and tailored gene delivery methods are needed for FPF.
• Adaptive platform trials such as the proposed REMAP-ILD could facilitate efficient simultaneous or sequential testing of multiple candidate therapies in subgroups of patients with specific mutations. This type of platform could create a drug discovery and testing pipeline for FPF drawing from repurposed or novel drugs.
• Identification and testing of new effective treatments and cure for FPF could be accelerated by collaboration with key stakeholders, including patients, patient advocacy and professional representative groups, regulatory agencies, academia, industry, and the broader medical community.

An expert consensus was developed at the meeting and in subsequent discussions during the writing of this Position Paper regarding the key questions relating to developing precision treatments for FPF. These questions included: what is the role of patient representatives and physician bodies, how can we generate and validate new preclinical models, what is the role of disease registries and biobanks, and what are the legal and ethical considerations in sharing patient samples. Issues also discussed were: new candidate experimental therapies and modes of delivery to the lung, a description of the existing clinical studies in FPF, and future international adaptive platform clinical trials. The aim of this paper is to provide an overview of evidence relating to these central issues, to provide an outline of current practice, and to highlight priority areas for research.

## Development of recommendations

The expert group first convened in Dublin, Ireland, in May 2022 for the COST Open-ILD Training School and conference. The overall theme of this meeting was to identify a pathway to discover and test new treatments for FPF with the intention of finding areas of consensus as well as areas in which there are knowledge gaps and insufficient evidence to draw clear conclusions. The conference started with an introductory lecture focusing on existing preclinical models of FPF and future models that are in development prepared in advance of the meeting and presented by K.H. with a subsequent question-and-answer session. There then followed a series of lectures and a larger, wide-ranging group discussion between all named authors, in which aspects of diagnosis, treatment and management of FPF were further discussed and published data were scrutinised. Priority areas were identified for discussion with a view to reaching consensus at the meeting. These included involvement of patients in research (D.C.), improving preclinical models using patient-specific induced pluripotent stem cells (iPSCs) (K.D.A.), improvement in the diagnosis and treatment of FPF in children (N.N.) and adults (R.B.), integrating patient registries in preclinical research (M.G. and M.K.), novel delivery methods for lung gene therapy (D.G.) and adaptive platform trials in ILD and FPF (G.J.). The views discussed at the meeting were further developed, including an update of the literature, as the manuscript was written and revised. This Position Paper represents a distillation of the views of the COST Open-ILD working group (Panel 1).

*Panel 1* **Summary statements developed by workshop participants: rationale for precision treatments in familial pulmonary fibrosis.**

• FPF is difficult to diagnose; genetic screening of patients and family members for causative gene mutations is complex requiring experts in the fields of clinical genetics, bioinformatics and respiratory medicine.
• Heterogeneity in disease onset and severity is a recognised feature of FPF and may be explained by incomplete genetic penetrance or other patient factors such as environmental exposures. This heterogeneity can influence response to therapy, yet remains poorly understood, therefore a better understanding of this phenomenon is essential so we can identify new treatments and minimise side effects and harm.
• Many current treatments lack an evidence base and include repurposed medications, anti-fibrotic and novel gene mutation specific treatments. In addition, mortality from FPF is high and a better understanding of the pathways that are involved in disease pathogenesis is required.

## What is familial pulmonary fibrosis?

In adults, ILD is a group of often fatal lung diseases caused by inflammation and fibrosis of the lung parenchyma. In its most severe form it can lead to respiratory failure and death often within three years of diagnosis.^3^ Genetic factors may cause or contribute significantly to the risk of developing ILD in adults. Indications for genetic screening were recently discussed in detail in both the recent European Respiratory Society (ERS) Statement on FPF and in the Pulmonary Fibrosis Foundation Perspective on Genetic Testing and are outlined in Panel 2.^4^^,^^5^ Patients with monogenic forms of ILD may have a worse prognosis than sporadic ILD, respond poorly to current treatments and some individuals may have serious adverse reactions to immunosuppression after transplantation.^6^, ^7^, ^8^ Whilst there are currently no evidence-based recommendations for specific treatments for FPF, current treatment practices are detailed in the ERS statement on FPF.^4^ These treatments are similar to those for patients with progressive pulmonary fibrosis, and are detailed in Panel 2. However, approved antifibrotic medications do have significant side effects and hepatic toxicity which often lead to discontinuation by the patient.^9^^,^^10^

*Panel 2* **Current diagnostic and treatment practices in familial pulmonary fibrosis.**

**Current indications for genetic analysis**

• Any paediatric patient with unexplained ILD.4^,^5
• Any patient with fibrotic ILD and one or more first or second-degree family members with fibrotic ILD.4^,^5
• Any patient with a relative carrying a pathogenic/likely pathogenic ILD variant.4^,^5
• Any patient with suspected short telomere syndrome∗4
• Any patient with short telomere length, where telomere length is performed before genetic testing.5
• Any patient with an idiopathic fibrosing ILD before the age of 50.4
• Genetic test results should be communicated with the patient with the help of a clinical geneticist or genetic counsellor.4^,^5

∗Short telomere syndrome includes pulmonary fibrosis, haematological and hepatic diseases.

^4^ European Respiratory Society Statement on Familial Pulmonary Fibrosis.

^5^ Pulmonary Fibrosis Foundation Perspective on the Role of Genetic Testing in Pulmonary Fibrosis.

**Current treatment practice in FPF**

• Patients should receive anti-fibrotic treatment as with all patients with progressive disease.
• Consideration of lung transplantation where deemed appropriate.
• In cases of short telomere syndrome, post-transplant immunosuppressive regimen should be carefully monitored and adjusted due to increased risk of significant side effects.
• In FPF in childhood or chILD, treatment is based on expert opinion and children should be offered therapeutic trials when available.
• Antifibrotic therapy with nintedanib has been shown to be safe and well tolerated in chILD.

Interstitial lung disease in children (chILD) is a heterogeneous collection of more than 200 rare and severe lung disorders affecting the distal parenchyma, often caused by monogenic mutations and can lead to death at birth or within the first years of life. ChILD can be self-limiting and is also associated with rare genetic causes of autoinflammatory disorders. While new anti-fibrotic medications offer hope of slowing disease progression in adults,^11^^,^^12^ no proven effective treatments are available for children, and lung transplant is the only effective treatment. ChILD and adult FPF may have very different pathogenesis, prognosis, and treatment. However, in this position paper for the purposes of identifying a unified approach to research we discuss the similar genetic variants that cause disease, similarities in preclinical models that are needed, and common methods to collect data from adults and children with ILD in international cohorts. We also recognise that patients with chILD now survive into adulthood and benefit from personalised management strategies in expert centres.

**Fig. 1:**
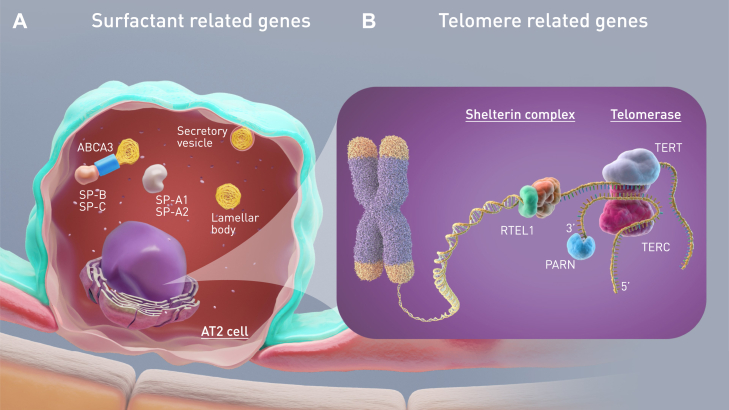
**Gene mutations and disease pathways found in familial pulmonary fibrosis (FPF)**: (**A**) Surfactant is a mixture of phospholipids and proteins produced by type 2 alveolar epithelial (AT2) cells, which prevents alveolar collapse at the end of expiration. The lipidic transporter ATP-binding cassette family A member 3 (ABCA3) is involved in the transport and storage of surfactant proteins (SP) into lamellar bodies. Secreted SP and phospholipids form the surfactant film at the air-liquid interface of the alveolar space. To date, in chILD, mutations have been described in genes encoding SP-C (*SFTPC*), ABCA3 (*ABCA3*), TTF1 (*NKX2-1*), SP-B (*SFTPB*), and in adult FPF, in genes encoding SP-A (*SFTPA1* and *SFTPA2*), SP-C, ABCA3 and TTF1. (**B**) Telomeres are nucleoprotein structures at the ends of linear chromosomes which protect genomic DNA. The activation of telomerase, composed of TERT and its RNA component TERC, prevents the attrition of chromosome ends during cell replication. Besides the telomerase complex, telomere homeostasis also requires the shelterin complex. In patients with FPF, mutations in the genes encoding telomere components, the most frequently identified being in *TERT, TERC*, *PARN* and *RTEL1*, result in alterations in telomere homeostasis and shortened telomeres in cells, such as AT2 cells.

Across the world diverse and rare mutations may cause FPF. In adults, loss-of-function telomere related gene (TRG) mutations are currently the most frequent identifiable monogenic cause (35–45%) and associated with 5–10% of all ILD (Fig. 1).^4^^,^^13^ Surfactant related gene (SRG) mutations have been shown to be the second most common identifiable cause of FPF in adults (5%),^14^ and the most common cause in children (20%).^15^ Patients present with heterogeneous phenotypes ranging from severe and fatal neonatal respiratory distress (surfactant protein (SP)-B and the intracellular transporter ABCA3) to chILD (SP-C, ABCA3) or adult forms of FPF (SP-C, ABCA3) with or without adenocarcinoma of the lung (SP-A1, SP-A2) (Fig. 1)^16^, ^17^, ^18^, ^19^ This Position Paper will not specifically detail the genetic causes of ILD that have notably been the subject of a recent ERS statement,^4^ but rather highlight and discuss what could be the future direction of *in vitro* studies and clinical trials for patients and their relatives. However, it is important to highlight that the rarity of individual variants contributes to challenges in diagnosis and the assistance of an expert genetic multidisciplinary team discussion can aid in an accurate diagnosis.^20^ The rarity of certain variants can also lead to a lack of basic mechanistic studies and randomised controlled trial data on effectiveness of treatments. Consequently, management strategies derived from other diseases are based on physicians’ experience and remain controversial.

## How can patients help to find precision treatments for FPF?

Patients, their families and their representative organisations already have established roles within the research and regulatory environment of ILD. Indeed, patients have contributed greatly to the generation of milestone documents such as Joint ERS/ATS/JRS/ALAT publications which includes the 2018 guidelines & 2022 update on Diagnosis and Treatments of IPF.^21^^,^^22^ Of particularly relevance to this Position Statement, patients were centrally involved in the drafting of the recently published ERS Statement on FPF.^4^ In addition, patients and their representative organisations such as EU-IPFF are key to the development of patient tools such as Patient Reported Outcome Measures (PROMs) and Patient Reported Experience Measures (PREMs). They also work closely with researchers on data collection via surveys and focus groups to further knowledge and to release patient originated findings. Significant potential exists for their contribution to the design, oversight and implementation of academic, preclinical and/or clinical studies, and policy documents which ultimately contribute towards the goal of precision treatments for FPF.

Our working group of experts included a patient (DC) with FPF who described patient opinions on precision treatments for FPF. He emphasised the importance of close collaboration of basic, translational, and clinical researchers with patients. He also stressed the benefit of providing patients with clear communication about their work, including clear lay descriptions of their research aims from the outset, formal regular updates on their research to patients and the public, and finally, explanations of the final results of research projects directly to patients He felt that this continuous process of communication with patients can foster trust and can lead to further important support for the international collaborative research effort that is needed to find new treatments for FPF.

## The role of registries and biobanks in developing new insights in pathogenesis and drug discovery

### Linking clinical cohorts

FPF comprises a collection of individually rare conditions with a similar phenotype, which cannot easily be filtered from the broad spectrum of diffuse parenchymal lung diseases. Therefore, robustly collecting clinical information and associated biomaterials from patients is crucial for investigating clinical course, pathophysiology and treatment options of such conditions.

Beyond informing on the natural history of specific FPF diagnoses, and the building of cohorts of FPF, these collections may contribute to disease classification,^23^ and serve as important and efficient tools to assess diagnostic and therapeutic needs of FPF with many recent newly discovered examples in children and adults.^24^, ^25^, ^26^, ^27^, ^28^ These registries could be used to assess the feasibility of clinical trials of repurposed or newly discovered drugs in ultra-rare conditions.^29^ In addition, the availability of collected biomaterials including DNA, RNA, plasma, tissue and immortalised or patient-derived iPSCs from a significant number of patients with monogenic FPF could facilitate studies of the molecular and cellular origins of disease.

There are several successful examples of national and international registries for ILD, including the recently launched euILDreg. This project aims to link both adult and paediatric registries, which are relatively scarce.^30^ In addition, it aims to include the Australasian Registry Network for Orphan Lung Disease (ARNOLD), the French register “RespiRare”, the United States chILD network, and the European chILD-EU register and biobank.^31^

Countries outside of Europe and in particular in developing countries could be better linked with researchers in Europe through a number of scientific networks. For example the chILD COST Action and the European Research Collaboration for chILD are broad networks of scientists, physicians and patient stakeholders which have membership across Europe but also in Turkey, Brazil, Canada and the USA. In addition, the European Management Platform for ILD is an open access online platform where physicians globally can contribute to a registry of patients with chILD and seek diagnostic support. Further linking of these European based networks with physician and scientific networks in Asia, Africa, and South and Central America could allow the sharing of genetic and phenotypic information about rare and common variants encountered in adults and children with FPF.

### Legal and ethical challenges

The primary legal challenge to linking biobank's or registries globally, is the lack of an overarching law governing biomedical research when using human samples and data, making it difficult to identify and comply with the applicable legal and ethical provisions within each national framework.^32^ For example, this is particularly true when attempting to share data from European citizens in the United States due to the application of General Data Protection Regulation 2016 (GDPR). Several attempts between the EU and USA have failed to reach agreement on sharing personal data between the two jurisdictions, limiting the data which can be shared. Even within the EU, GDPR specification clauses permits Member States scope when applying certain aspects of the GDPR, resulting in different participant information leaflets (PIL) and consent forms in different jurisdictions based on language, national law and local Research Ethic Committee requirements which can all delay the initiation of research.

Strategies to improve global sharing of data and samples within the parameters of current regulation could involve: a) identifying legal and ethical commonalities to allow for the development of standardised PIL and consent templates with sections allowing for differing national legal requirements; b) the development of a tool kit for researchers to understand differing legal and ethical requirements in participating countries; and c) a clear data flow and management plan. At the EU Governmental level the proposed European Health Data Space Regulation (EHDS) should in the future help to harmonise the law in relation to the use of health data.

Despite these challenges, linking registries and biobanks across Europe and the globe are central for future treatments and cure of FPF.

## Animal and cell based preclinical models of ILD

### Existing preclinical models of FPF

Currently, exposure of mice to bleomycin is the main preclinical model used to explore the pathogenesis of and test new drugs for pulmonary fibrosis including IPF.^33^ However, this model is limited as the fibrosis induced does not progress, can resolve after exposure and thus fails to recapitulate the human disease. Consequently, the model typically does not predict clinical efficacy of candidate therapeutics in humans.^33^ A growing literature now implicates epithelial dysfunction as an initiating event in ILD, with a number of variants in gene loci expressed in lung epithelia associated with sporadic ILD and FPF.^34^^,^^35^ Type 2 alveolar epithelial (AT2) cell dysfunction, in particular, has been repeatedly implicated in the pathogenesis of chILD, FPF, and sporadic ILD.^36^, ^37^, ^38^, ^39^ Based on this new understanding more specific models of FPF are emerging,^40^, ^41^, ^42^ for example a recently developed transgenic mouse model of monogenic lung fibrosis, based on the most common surfactant protein C mutation (*SFTPC*^I73T^).^43^ Unlike the bleomycin model, this mouse model develops spontaneous acute alveolitis with overexpression of pulmonary fibrosis biomarkers as well as fibrotic remodelling closer to ILD pathogenesis in humans.

Mouse models have also been used to model pulmonary fibrosis due to telomere shortening in an attempt to explore the consequences of alveolar stem cell failure. However, modelling short telomere related pulmonary fibrosis in laboratory mice is challenging as they have extremely long telomeres,^44^ and therefore in the absence of the enzyme telomerase, telomere dysfunction can be produced only after several generations of breeding, preventing cell type-specific studies.^45^ Despite these limitations, Alder et al. tested the regenerative capacity of AT2 and stromal cells from mice with short telomeres and found decreased alveolar organoid colony formation and growth.^46^ They also found that AT2 cells of mice with conditional deletion of shelterin related genes survived but remained dormant, expressed the gene and protein signature of cellular senescence, and triggered an immune response consistent with inflammation in the lung. These mice uniformly died after challenge with bleomycin. In a further study, mice treated with the telomerase gene, *Tert* using adeno-associated vectors have shown amelioration of fibrosis through telomere elongation and increased proliferation of AT2 cells leading to reduced DNA damage, apoptosis and senescence burden.^47^ However, these experimental approaches may have limited clinical potential as they are not human-specific and the preclinical model is not suitable for high-throughput screening.

### Emerging preclinical models of FPF

Since there are significant differences between murine and human ILD, there is a pressing need for reliable human preclinical disease models that would not only provide further insight into the pathophysiology of human AT2 cell dysfunction at ILD inception but would also serve as drug testing platforms. Over the past decade, methods have been developed for the derivation of lung lineages from pluripotent stem cells (PSCs), or their engineered equivalents, iPSCs,^48^, ^49^, ^50^, ^51^, ^52^, ^53^ providing new alternative cell systems that can be harnessed to develop *in vitro* disease models, drug screens, or regenerative therapies for difficult-to-treat lung diseases, such as ILD. Importantly, iPSCs are genetically identical to the individual from whom they are derived, raising the prospect of utilising iPSCs as patient-specific *in vitro* disease models for predicting the effectiveness of precision therapeutics.

Several recent studies have demonstrated proof-of-concept that lung epithelia derived from human PSCs carrying certain mutations could model different aspects of the AT2 cell dysfunction present in ILD.^54^, ^55^, ^56^, ^57^, ^58^ For example, iPSC-derived AT2 (iAT2) cells have been applied to model dysfunction of lysosome-related organelles, such as the lamellar bodies of AT2 cells, in the setting of mutations in Hermansky-Pudlak syndrome genes,^54^^,^^55^ and AT2 cell senescence, a feature of ILD, driven by TRG mutations.^58^ These patient-derived models may in the future be utilised to assess the pathogenicity of rare variants.

Patient-specific iPSCs carrying an AT2 cell-exclusive pathogenic variant (*SFTPC*^*I73T*^) have also been applied to study the intrinsic epithelial dysfunction at the inception of ILD.^57^ In this study, *SFTPC* mutant iAT2 cells demonstrated pro-SFTPC protein mistrafficking and misprocessing with resultant diminished alveolar progenitor capacity, proteostasis perturbations, mitochondrial dysfunction, metabolic reprogramming from oxidative phosphorylation to glycolysis, and inflammatory activation. In a proof-of-principle experiment, the application of this model system as a preclinical human disease drug testing platform was assessed by testing the effect of hydroxychloroquine, a medication commonly used in paediatric patients with chILD, on *SFTPC*^*I73T*^-expressing cells. Furthermore, the *in vivo* validation of key *in vitro* observations in this study, in donor lung explant sections and the *Sftpc*^*I73T*^ genetic mouse model,^43^ further supports the concept that PSC-derived lung epithelia can be used as effective preclinical human models for disease modelling and drug testing.

Future work would require increasing the complexity of the available PSC-model systems by including additional lineages (e.g. mesenchyme, immune lineages) to allow for better examination of the cellular interactions responsible for disease initiation and progression. Careful characterisation of the newly developed models and benchmarking against human samples and mouse models would be equally important.

## Clinical trials in FPF

### Clinical studies in TRG mutation associated ILD

Synthetic androgen therapy is already an experimental therapy in TRG-associated disease with historic animal and human trials and ongoing clinical trials.^1^^,^^59^^,^^60^ Initially, androgen therapy with danazol was shown to be an effective treatment for bone marrow failure associated with TRG mutations with a 69–83% haematological response and increased telomere length.^1^^,^^61^ However, in these studies almost 1/3 of patients stopped treatment within 2 years mainly due to hepatic toxicity. Data about the effect of danazol in ILD patients were limited, but suggested a reduced decline of lung function.^59^ More recently, a retrospective study by Hoffman et al., suggests that danazol is unlikely to be effective in patients with advanced IPF.^62^ However, patients recruited to this study had very severe pulmonary fibrosis with a weak association to genetic factors. These studies suggest that patients may need to be treated with androgens for several years, at earlier time points and with less toxic androgens such as nandrolone to reduce fibrosis progression.^59^ Indeed, two prospective clinical trials with danazol dedicated to patients with ILD, TELO-SCOPE (NCT04638517) and ANDROTELO (NCT03710356) are ongoing (Table 1) and should reveal important data about safety and efficacy of androgen therapy for patients with ILD.

**Table 1:** Ongoing clinical trials in familial pulmonary fibrosis.

Name/NCT Number	Aim	Method	Population	Intervention	Primary outcome	Enrolment	Start/End
ANDROTELO NCT03710356	Assess the safety and efficacy of danazol in patients with severe haematological or pulmonary disease related to telomeropathies.	Multi-centre Bayesian trial.	Subjects aged ≥15 years with mutation of a gene involved in telomere maintenance and severe haematological involvement and/or ILD.	Danazol for 12 months.	Haematological response or pulmonary response at 12 months.	40	2018-10-20 2022-10-20
TELO-SCOPE NCT04638517	Assess the safety and efficacy of danazol in adults and children with ILD associated with telomere shortening.	Multi-centre, double-blind, placebo-controlled, randomised trial.	Subjects aged >5 years with ILD associated with age-adjusted telomere length ≤10th centile.	Danazol or Placebo for 12 months.	Change in absolute telomere length in base pairs at 12 months.	50	2021-09-07 2025–06
EXG34217 Study NCT04211714	Assess the safety and tolerability of EXG34217 in bone marrow failure patients with telomere biology disorders	Open label, single centre study	Age >18 years, with mild or moderate bone marrow failure and a diagnosis of telomere biology disorders.	Single infusion of autologous CD34^+^ cells contacted *ex vivo* with EXG-001.	Number of participants with treatment-related adverse events from baseline to 12 months.	12	2021-04-08 2027-04-08

Abbreviations: NCT, National Clinical Trail; ILD, interstitial lung disease.

Potential new targeted treatment for patients with TRG mutations raise specific challenges which will need to be addressed before safe and effective treatments are available for patients. Firstly, we anticipate that telomere directed drug treatments will have a narrow therapeutic range as increased telomerase activity is associated with tumour growth and cancer.^63^ In particular, treating relatives (asymptomatic carriers of the mutation) for years with such drugs raises significant safety concerns. Secondly, multiple diverse TRG mutations have been associated with FPF, therefore given the diversity of mutations, treatment strategies for patients with TRG mutations will require interventions that either address the whole telomere pathway or the development of multiple medications that are stratified by mutation.

The most obvious type of potential targeted treatment for TRG mutations is gene therapy. However, there are very limited gene therapy strategies currently available despite repeated efforts in this area in animal models.^64^, ^65^, ^66^ In mice, nebulized and intravenous delivery of *TERT* therapy via high-capacity cytomegalovirus virus (CMV) vector have been developed and show promise in prolonging telomere length and longevity,^67^ but there are many technical and safety challenges in translating this type of technology to humans. Despite these challenges a clinical trial is currently recruiting patients with TRG mutations with bone marrow failure. In this trial, *ex vivo* telomere elongation is accomplished in autologous CD34+ hematopoietic stem cells using gene therapy before cells are later returned to patients. Preliminary results have shown potential emergence of a cell population with longer telomeres *in vivo* without acute or late-stage complications (NCT04211714).

Finally, the recent history of drug development in cystic fibrosis (CF) encourages us to seek a treatment specifically targeting the pathway impaired by the germline loss of function. A promising candidate for TRG defects is a poly(A) polymerase 5 (PAPD5) inhibitor, which has shown promise in *in vitro* and *in silico* models restoring telomerase activity in patient stem cells.^68^ This new approach will need further investigation in future phase 1 and 2 clinical studies. Additionally, other emerging therapeutic possibilities include RNA therapy, given the recent advancements in mRNA technology in COVID-19 vaccines.^69^ *In vivo* studies are needed to define the cellular target, and the type of RNA to deliver, however, RNA therapy has the potential to answer many of the specific problems raised by TRG mutations outlined above.

### Clinical studies and emerging agents in SRG mutation ILD

To date, only non-specific treatments are available for SRG mutation ILD, such as corticosteroids, azithromycin, hydroxychloroquine.^70^^,^^71^ However, these drugs lack proven efficacy and the prognosis of chILD remains poor, often leading to terminal respiratory insufficiency and requiring lung transplantation. Specific treatments are urgently needed. In addition, due to the rarity of affected children, few clinical trials have been completed in chILD. The first chILD clinical trial performed over a four year period, to assess the efficacy of hydroxychloroquine with a start or stop protocol in 35 patients failed to show any difference between groups, being limited by low numbers of recruited patients, the heterogeneous forms of chILD included and the short duration of the treatment (four weeks).^29^ In 2022, the first international double-blind, placebo-controlled study in chILD aimed to assess the dose-exposure and safety of the anti-fibrotic, nintedanib over 24 weeks of treatment in 29 children aged 6–17 with fibrosing ILD and clinically significant disease.^72^ Using a weight-based dosing regimen in patients with chILD, comparable exposure to adults and an acceptable safety profile were found. However, patients did have significant adverse events including diarrhoea.

### Delivering novel gene therapies to patients

Lethal surfactant deficiencies presenting as severe respiratory distress in newborns can be due to recessive mutations in the *SFTPB* and *ABCA3* genes. Gene addition therapy to restore surfactant production offers a potential cure.^73^ Since the first demonstration of increased survival of SP-B deficient mice after delivery of mRNA to the lungs,^74^ several improved gene therapy vectors have been evaluated. Most recently, the use of recombinant AAV to express SP-B in the target AT2 cells extended survival of these mice, as well as restoring lamellar body morphology.^75^ Integrating lentiviral vectors may further enhance this approach since the vector genome will be retained in the daughter cells from subsequent cell divisions leading to longer-term transgene expression. A lentivirus encoding the *SFTPB* cDNA has been shown to correct the SP-B deficiency phenotype in a cell culture model that recapitulates key characteristics of human AT2 cells in primary culture.^76^^,^^77^ An additional advantage of lentiviral vectors is their greater capacity to package large transgene sequences compared with AAV vectors, such as the sequences (promoter and transgene) required for expression of the large, full length *ABCA3* cDNA for treatment of ABCA3-deficiency. Although full surfactant production restoration in the lungs is the best possible gene therapy outcome, even stabilisation of the condition may offer a window in which other treatment options can be explored. The successful experiences in making gene therapy available for spinal muscular atrophy,^78^ provide hope that such an approach is also possible in FPF.

### New clinical trial designs for studies in FPF

The key to successful research programs in rare diseases is through multicentre collaboration. This collaboration is crucial for both *in vitro* experimental studies and clinical trials, especially when the drugs being considered for testing are only likely to have modest effects, and the number of patients required is likely to be large. One approach to enriching populations is to stratify patients by mechanism, for example telomeropathies. However, as TRG variants are identifiable in less than 10% of all ILD cases this will reduce the number of patients available, which may mitigate the increased power associated with a more homogenous population. It is therefore crucial that future clinical trials are maximised for efficiency and can be undertaken by international consortia. This is the purpose of the Randomised Embedded Multifactorial Adaptive Platform in Interstitial Lung Diseases (REMAP-ILD), which will use Bayesian statistical models to incorporate all data into adaptive decision making during the running of the trial, facilitating early stopping due to futility and concentrating the randomisation of patients to interventions that are likely to be beneficial. This type of trial has shown promise in other diseases such as community-acquired pneumonia (REMAP-CAP).^79^

**Fig. 2:**
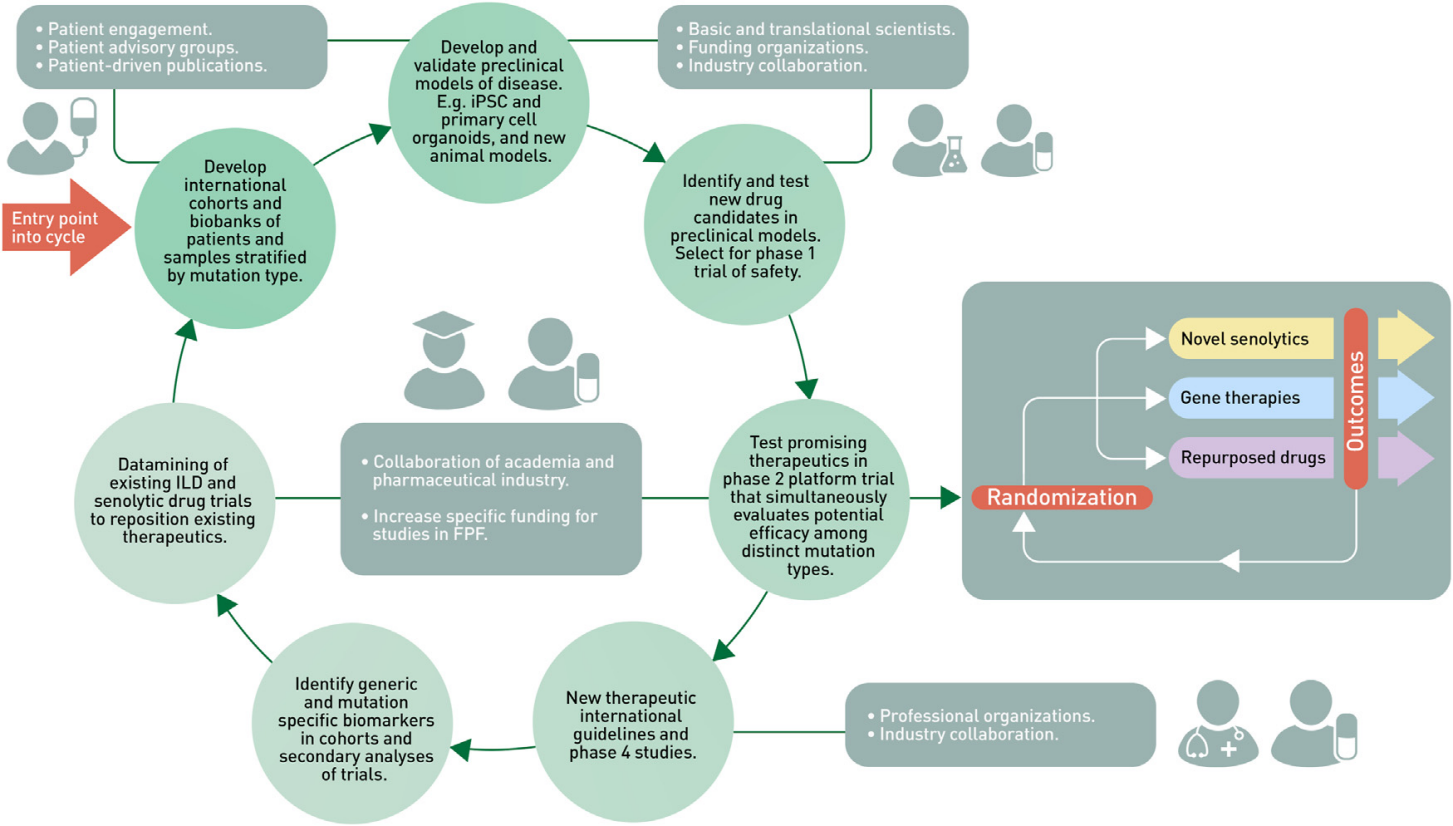
**Overview of framework to develop precision treatments for familial pulmonary fibrosis**: The figure outlines the proposed engagement of people, resources and processes required to identify and test new therapeutics for familial pulmonary fibrosis (FPF). Future clinical trials could employ an adaptive platform design such as the proposed Randomised Embedded Multifactorial Adaptive Platform in Interstitial Lung Diseases (REMAP-ILD) to test multiple novel or repurposed pharmaceutical therapies in tandem. The European Respiratory Society promotes global chILD networking by funding and supporting the Clinical Research Collaboration for chILD-EU for over 10 years. Potential future funding sources for FPF registries and biobanking include the NIH's Rare Diseases Research Programs, the Horizon programme funding from the European Commission and the European Joint Programme in Rare Diseases. Collaborative efforts with pharmaceutical industries who may explore the repurposing of already approved medications for IPF, PFF and other lung diseases in addition to drug development companies who specialise in orphan drug development are crucial sources for support.

The proposed studies which include patient stratification by genotype or biomarkers (Fig. 2), emphasise the need to assess the genetics and telomere length in all patients with suspected FPF in clinical practice,^4^ as it is clear that identifying patients with causative mutations at the earliest opportunity is fundamental in delivering clinical trials in FPF. It is also important to point out that there are concerns about the harmful effects of immunosuppressants on people with shorter telomeres,^80^ therefore it is crucial that future clinical trials can stratify by TRG mutation and telomere length, and alternative strategies developed for these patients. One such class of therapeutics includes drugs which attempt to eliminate senescent cells (senolytics) and those that modulate the proinflammatory senescent secretome (senostatics). These drugs have shown promise in treating patients with pulmonary fibrosis including IPF.^81^^,^^82^ This new family of drugs may be expected to have enhanced effects in people with shorter telomeres or TRG mutations. However, given the relatively low proportion of patients with FPF who have identifiable TRG mutations or short telomeres, it will be crucial to establish international cohorts so that adequate numbers of patients can be recruited to clinical trials of these potential treatments.

## Conclusions and future directions

Recent genetic discoveries have led to significant advances in the understanding of ILD pathophysiology. However, these discoveries in large have not yet impacted on patient care. In addition, the detection of these mutations in patients leads to the need for genetic counselling and raises the question of pre-symptomatic assessment in asymptomatic relatives, an endeavour that lacks an evidence base given the lack of effective treatments for symptomatic and indeed preventative or pre-symptomatic disease. Next generation sequencing now allows us to identify distinct genetic subgroups of patients so we can match them with new and repurposed candidate therapies. However, phenotype heterogeneity even within the same genotype, may influence the outcomes of therapies. Therefore, there is now an urgent need for all stakeholders to develop a framework to coordinate joint efforts to advance our understanding of FPF disease mechanisms and identify and test new precision therapies.

A major challenge to developing new therapies is the rarity of these individual genetic conditions which may reduce the attractiveness of drug development by the pharmaceutical industry. But as the example of new CFTR corrector therapies in CF has shown us, developing new drugs for genotypes with common mechanisms has meant an explosion in the development of new successful treatments for CF, which has been transformational for patients. Indeed, CFTR corrector medications themselves may be repurposed treatments for FPF due to ABCA3 mutations.^83^ We believe that with broad stakeholder commitment, along with the global initiative that we have proposed in this Position Paper, the field can respond to the multiple challenges facing it and develop new precision treatments for patients with FPF (Panel 3).

*Panel 3* **Summary statements developed by participants: designing a research framework to develop precision treatments for familial pulmonary fibrosis.**

**The central role of patients and families in FPF research**

• Patients, their families and their representative organisations already have established roles within the research and regulatory environment. Significant potential exists for their contribution to the design, oversight and implementation of academic, clinical, research and policy documents and projects. Key areas for PPI include guidelines, funding consortiums, lay summaries and co-creation of patient facing materials, websites and packaging, all vital for the overall success and integrity of a given paper or project.
• Patients are key to the development of patient tools such as Patient Reported Outcome Measures (PROMs) and Patient Reported Experience Measures (PREMs) and are actively consulted on various aspects of both preclinical and clinical studies. They also work closely with researchers on data collection via surveys and focus groups to further knowledge and to release patient originated findings.

**Development and validation of new preclinical testing platforms**

• Robust preclinical models of FPF are lacking as there is limited access to tissue that faithfully recapitulates normal and abnormal human lung biology creating a major hurdle to developing new therapies. New preclinical models of FPF will aid our understanding of specific biological processes and identify key mechanistic pathways and future therapeutic targets. Validation of these novel *in vitro* and *in vivo* models with clinical outcomes in patients will need to occur before these platforms can be used to discover new therapies.

**The essential role of international clinical cohorts**

• Access to timely genetic screening of all children and adults with FPF is the first step in setting up meaningful cohorts in FPF.
• Efficient collection of clinical information and associated biomaterials from these subjects are crucial for investigating the clinical course, pathophysiology and treatment options for FPF. These registers and biobanks require appropriate data safety standards, ethics and regulatory approvals, long term financial, institutional and structural resources, standard operating procedures and an effective communication framework with all sites.
• International, multicentre, observational cohort studies that collect clinical, physiological, radiological, and biological data and samples in a harmonised way will facilitate deep phenotyping of patients and allow identification of mechanistic pathways.

**Adaptive platform clinical trials to advance precision medicine**

• Future clinical trials need to be maximised for efficiency and undertaken by international consortia. Adaptive platform trials such as Randomised Embedded Multifactorial Adaptive Platform in Interstitial Lung Diseases (REMAP-ILD), which will use Bayesian statistical models to incorporate all data into adaptive decision making during the running of the trial, will facilitate early stopping due to futility and will concentrate the randomisation of patients to interventions that are likely to be beneficial.

## Outstanding questions

In order to develop a successful framework to identify and test new precision treatments for FPF, we must answer a number of key unresolved questions, including; What are the key cells which drive FPF and molecular pathways disrupted by individual genetic mutations? Can patients with FPF be enrolled in trials that study sporadic ILD? Can gene stratified treatments for diverse genetic mutations in FPF be designed? Can biomarkers for early FPF be developed to facilitate screening and treatment of pre-symptomatic disease in high risk family members? Are there identifiable sub-phenotypes of FPF in patients with the same genetic mutation? What are robust surrogate outcome measures for FPF clinical trials? What are the highest priority therapeutic candidates for testing in precision medicine trials?

## Contributors

K.H. and R.B. conceptualised the Position Paper. K.H. acquired funding, administered the project, supervised and wrote the original draft, reviewed and edited the final manuscript. R.B., N.N. M.O., Q.P., M.K., M.G., G.J., D.G.,L.V.G., and K.D.A. co-wrote the original draft, validated, edited and reviewed the final manuscript. All authors read and approved the final version of the manuscript.

## Declaration of interests

No authors have stocks or shares, equity, a contract of employment, or a named position on a company board. KH reports grants from COST (European Cooperation in Science and Technology) Innovator Grant (CIG 16125) and the HRB Emerging Clinical Scientist Award (ECSA-2020-011) which supported the manuscript. KH also reports grants from Moderna Tx, and lecturer fees from Boehringer Ingelheim and PatientMPower outside of the submitted work. MO reports grants from the Irish Research Council and support for attending meetings from the Irish Thoracic Society and GlaxoKleinSmith outside of the submitted work. QP reports support for attending meetings from Janssen and lecturer fees from Gilead outside of the submitted work. LG reports a paid leadership role in the EU-IPFF and unrestricted organisational grants to EU-IPFF from Bristol Meyer Squibb, Boehringer Ingelheim, Chiesi Pharmaceuticals, Ferrar Pharmaceuticals, CSL Behring, Trevi Therapeutics, The Roche Group, Vicore Pharma. LG also reports support for attending meetings from the ERS, European Lung Foundation, European Reference Network on Rare Respiratory Diseases and unpaid leadership roles in European Reference Network on Rare Respiratory Diseases, European Lung Foundation, the Irish Lung Fibrosis Association. GJ reports grants from Astra Zeneca, Biogen, Galecto, GlaxoSmithKline, Nordic Biosciences, RedX, Pliant, consulting fees from Astra Zeneca, Brainomix, Bristol Myers Squibb, Chiesi, Cohbar, Daewoong, GlaxoSmithKline, Veracyte, Resolution Therapeutics, Pliant, and lecturer fees from Boehringer Ingelheim, Chiesi, Roche PatientMPower, AstraZeneca outside of the submitted work. GJ also reports payments from Pinsent Masons LLP for expert testimony, participation on advisory board for Boehringer Ingelheim, Galapagos, Vicore, and leadership roles for NuMedii and Action for Pulmonary Fibrosis. MG reports grants, participation on advisory and adjudication boards, and lecturer fees from Boehringer Ingelheim outside of the submitted work. NN reports grants from Million Dollar Bike Ride, Chancellerie des Universités: Legs Poix, (n°2022000594), support for attending meetings from the ERS and an unpaid leadership role on the ERS chILDEU CRC. RB reports consulting fees from Boehringer Ingelheim, Ferrer, and Sanofi and lecturer fees from Boehringer Ingelheim and Roche outside of the submitted work. RB also received support for attending meetings from Boehringer Ingelheim, Roche and Chiesi, and participation on advisory boards for Savara.

## References

[1] Townsley D.M., Dumitriu B., Young N.S. (2016). Danazol treatment for telomere diseases. N Engl J Med.

[2] Mackintosh J.A., Pietsch M., Lutzky V. (2021). TELO-SCOPE study: a randomised, double-blind, placebo-controlled, phase 2 trial of danazol for short telomere related pulmonary fibrosis. BMJ Open Respir Res.

[3] Travis W.D.C.U., Hansell D.M., King Jr TE. (2013). An official American Thoracic Society/European Respiratory Society statement: update of the international multidisciplinary classification of the idiopathic interstitial pneumonias. Am J Respir Crit Care Med.

[4] Borie R., Kannengiesser C., Antoniou K. (2022). European respiratory society statement on familial pulmonary fibrosis. Eur Respir J.

[5] Newton C.A., Oldham J.M., Applegate C. (2022). The role of genetic testing in pulmonary fibrosis: a perspective from the pulmonary fibrosis foundation genetic testing work group. Chest.

[6] Garcia-Sancho C.B.-R.I., Fernandey-Plata M.R., Navarro C. (2011). Familial pulmonary fibrosis is the strongest risk factor fo idiopathic pulmonary fibrosis. Respir Med.

[7] Cutting C.C.B.W., Dao N., Pugashetti J.V., Garcia C.K., Oldham J.M., Newton C.A. (2021). Family history of pulmonary fibrosis predicts worse survival in patients with interstitial lung disease. Chest.

[8] Krauss E., Gehrken G., Drakopanagiotakis F. (2019). Clinical characteristics of patients with familial idiopathic pulmonary fibrosis (f-IPF). BMC Pulm Med.

[9] Crestani B., Huggins J.T., Kaye M. (2019). Long-term safety and tolerability of nintedanib in patients with idiopathic pulmonary fibrosis: results from the open-label extension study, INPULSIS-ON. Lancet Respir Med.

[10] Cottin V., Koschel D., Gunther A. (2018). Long-term safety of pirfenidone: results of the prospective, observational PASSPORT study. ERJ Open Res.

[11] Noble P.W.A.C., Bradford W.Z., Costabel U. (2011). Pirfendione in patients with idiopathic pulmonary fibrosis (CAPACITY): two randomised trials. Lancet.

[12] Richeldi L. dBR., Raghu G., Azuma A. (2014). Efficacy and safety of nintendanib in idiopathic pulmonary fibrosis. N Engl J Med.

[13] Alder J.K., Sutton R.M., Iasella C.J. (2022). Lung transplantation for idiopathic pulmonary fibrosis enriches for individuals with telomere-mediated disease. J Heart Lung Transplant.

[14] Garcia C.K. (2011). Idiopathic pulmonary fibrosis: update on genetic discoveries. Proc Am Thorac Soc.

[15] Torrent-Vernetta A., Gaboli M., Castillo-Corullon S. (2022). Incidence and prevalence of children's diffuse lung disease in Spain. Arch Bronconeumol.

[16] Wambach J.A., Casey A.M., Fishman M.P. (2014). Genotype-phenotype correlations for infants and children with ABCA3 deficiency. Am J Respir Crit Care Med.

[17] Nogee L.M., Garnier G., Dietz H.C. (1994). A mutation in the surfactant protein B gene responsible for fatal neonatal respiratory disease in multiple kindreds. J Clin Invest.

[18] Cottin V., Cordier J.F. (2011). SFTPC mutations in patients with familial pulmonary fibrosis: combined with emphysema?. Am J Respir Crit Care Med.

[19] Legendre M., Butt A., Borie R. (2020). Functional assessment and phenotypic heterogeneity of SFTPA1 and SFTPA2 mutations in interstitial lung diseases and lung cancer. Eur Respir J.

[20] Borie R., Kannengiesser C., Gouya L. (2019). Pilot experience of multidisciplinary team discussion dedicated to inherited pulmonary fibrosis. Orphanet J Rare Dis.

[21] Raghu G., Remy-Jardin M., Myers J.L. (2018). Diagnosis of idiopathic pulmonary fibrosis. An official ATS/ERS/JRS/ALAT clinical practice guideline. Am J Respir Crit Care Med.

[22] Raghu G., Remy-Jardin M., Richeldi L. (2022). Idiopathic pulmonary fibrosis (an update) and progressive pulmonary fibrosis in adults: an official ATS/ERS/JRS/ALAT clinical practice guideline. Am J Respir Crit Care Med.

[23] Griese M. (2022). Etiologic classification of diffuse parenchymal (interstitial) lung diseases. J Clin Med.

[24] Vavassori S., Chou J., Faletti L.E. (2021). Multisystem inflammation and susceptibility to viral infections in human ZNFX1 deficiency. J Allergy Clin Immunol.

[25] Magg T., Okano T., Koenig L.M. (2021). Heterozygous OAS1 gain-of-function variants cause an autoinflammatory immunodeficiency. Sci Immunol.

[26] Schuch L.A., Forstner M., Rapp C.K. (2021). FARS1-related disorders caused by bi-allelic mutations in cytosolic phenylalanyl-tRNA synthetase genes: look beyond the lungs. Clin Genet.

[27] Xu Z., Lo W.S., Beck D.B. (2018). Bi-Allelic mutations in phe-tRNA synthetase associated with a multi-system pulmonary disease support non-translational function. Am J Hum Genet.

[28] Rapp C.K., Van Dijck I., Laugwitz L. (2021). Expanding the phenotypic spectrum of FINCA (fibrosis, neurodegeneration, and cerebral angiomatosis) syndrome beyond infancy. Clin Genet.

[29] Griese M., Kappler M., Stehling F. (2022). Randomized controlled phase 2 trial of hydroxychloroquine in childhood interstitial lung disease. Orphanet J Rare Dis.

[30] Guenther A., European I.P.F.N. (2011). The European IPF Network: towards better care for a dreadful disease. Eur Respir J.

[31] Griese M., Seidl E., Hengst M. (2018). International management platform for children's interstitial lung disease (chILD-EU). Thorax.

[32] Kaye J., Briceno Moraia L., Curren L. (2016). Consent for biobanking: the legal frameworks of countries in the BioSHaRE-EU project. Biopreserv Biobank.

[33] Carrington R., Jordan S., Pitchford S.C., Page C.P. (2018). Use of animal models in IPF research. Pulm Pharmacol Ther.

[34] Kropski J.A., Blackwell T.S., Loyd J.E. (2015). The genetic basis of idiopathic pulmonary fibrosis. Eur Respir J.

[35] Garcia C.K. (2018). Insights from human genetic studies of lung and organ fibrosis. J Clin Invest.

[36] Katzenstein A.L. (1985). Pathogenesis of "fibrosis" in interstitial pneumonia: an electron microscopic study. Hum Pathol.

[37] Barkauskas C.E., Noble P.W. (2014). Cellular mechanisms of tissue fibrosis. 7. New insights into the cellular mechanisms of pulmonary fibrosis. Am J Physiol Cell Physiol.

[38] Selman M., Pardo A. (2014). Revealing the pathogenic and aging-related mechanisms of the enigmatic idiopathic pulmonary fibrosis. an integral model. Am J Respir Crit Care Med.

[39] Winters N.I., Burman A., Kropski J.A., Blackwell T.S. (2019). Epithelial injury and dysfunction in the pathogenesis of idiopathic PulmonaryFibrosis. Am J Med Sci.

[40] Katzen J., Wagner B.D., Venosa A. (2019). An SFTPC BRICHOS mutant links epithelial ER stress and spontaneous lung fibrosis. JCI Insight.

[41] Yao C., Carraro G., Konda B. (2017). Sin3a regulates epithelial progenitor cell fate during lung development. Development.

[42] Rodriguez L., Tomer Y., Carson P. (2022). Chronic expression of a clinical SFTPC mutation causes murine lung fibrosis with IPF features. Am J Respir Cell Mol Biol.

[43] Nureki S.I., Tomer Y., Venosa A. (2018). Expression of mutant Sftpc in murine alveolar epithelia drives spontaneous lung fibrosis. J Clin Invest.

[44] Hemann M.T., Greider C.W. (2000). Wild-derived inbred mouse strains have short telomeres. Nucleic Acids Res.

[45] Blasco M.A., Lee H.W., Hande M.P. (1997). Telomere shortening and tumor formation by mouse cells lacking telomerase RNA. Cell.

[46] Aldera J.K., Barkauskas C.E., Limjunyawong N. (2015). Telomere dysfunction causes alveolar stem cell failure. Proc Natl Acad Sci U S A.

[47] Povedano J.M., Martinez P., Serrano R. (2018). Therapeutic effects of telomerase in mice with pulmonary fibrosis induced by damage to the lungs and short telomeres. Elife.

[48] Longmire T.A., Ikonomou L., Hawkins F. (2012). Efficient derivation of purified lung and thyroid progenitors from embryonic stem cells. Cell Stem Cell.

[49] Mou H., Zhao R., Sherwood R. (2012). Generation of multipotent lung and airway progenitors from mouse ESCs and patient-specific cystic fibrosis iPSCs. Cell Stem Cell.

[50] Dye B.R., Hill D.R., Ferguson M.A. (2015). In vitro generation of human pluripotent stem cell derived lung organoids. Elife.

[51] Gotoh S., Ito I., Nagasaki T. (2014). Generation of alveolar epithelial spheroids via isolated progenitor cells from human pluripotent stem cells. Stem Cell Rep.

[52] Hawkins F., Kramer P., Jacob A. (2017). Prospective isolation of NKX2-1-expressing human lung progenitors derived from pluripotent stem cells. J Clin Invest.

[53] Huang S.X., Green M.D., de Carvalho A.T. (2015). The in vitro generation of lung and airway progenitor cells from human pluripotent stem cells. Nat Protoc.

[54] Suezawa T., Kanagaki S., Korogi Y. (2021). Modeling of lung phenotype of Hermansky-Pudlak syndrome type I using patient-specific iPSCs. Respir Res.

[55] Korogi Y., Gotoh S., Ikeo S. (2019). In vitro disease modeling of hermansky-pudlak syndrome type 2 using human induced pluripotent stem cell-derived alveolar organoids. Stem Cell Rep.

[56] Strikoudis A., Cieslak A., Loffredo L. (2019). Modeling of fibrotic lung disease using 3D organoids derived from human pluripotent stem cells. Cell Rep.

[57] Alysandratos K.D., Russo S.J., Petcherski A. (2021). Patient-specific iPSCs carrying an SFTPC mutation reveal the intrinsic alveolar epithelial dysfunction at the inception of interstitial lung disease. Cell Rep.

[58] Fernandez R.J., Gardner Z.J.G., Slovik K.J. (2022). GSK3 inhibition rescues growth and telomere dysfunction in dyskeratosis congenita iPSC-derived type II alveolar epithelial cells. Elife.

[59] Cle D.V., Catto L.F.B., Gutierrez-Rodrigues F. (2023). Effects of nandrolone decanoate on telomere length and clinical outcome in patients with telomeropathies: a prospective trial. Haematologica.

[60] Bar C., Huber N., Beier F., Blasco M.A. (2015). Therapeutic effect of androgen therapy in a mouse model of aplastic anemia produced by short telomeres. Haematologica.

[61] Khincha P.P., Wentzensen I.M., Giri N., Alter B.P., Savage S.A. (2014). Response to androgen therapy in patients with dyskeratosis congenita. Br J Haematol.

[62] Hoffman T.W., van Moorsel C.H.M., van der Vis J.J., Biesma D.H., Grutters J.C. (2023). No effect of danazol treatment in patients with advanced idiopathic pulmonary fibrosis. ERJ Open Res.

[63] Lauriola A., Davalli P., Marverti G., Caporali A., Mai S., D'Arca D. (2022). Telomere dysfunction is associated with altered DNA organization in trichoplein/tchp/mitostatin (TpMs) depleted cells. Biomedicines.

[64] Derevyanko A., Whittemore K., Schneider R.P., Jimenez V., Bosch F., Blasco M.A. (2017). Gene therapy with the TRF1 telomere gene rescues decreased TRF1 levels with aging and prolongs mouse health span. Aging Cell.

[65] Povedano J.M., Martinez P., Flores J.M., Mulero F., Blasco M.A. (2015). Mice with pulmonary fibrosis driven by telomere dysfunction. Cell Rep.

[66] Bar C., Povedano J.M., Serrano R. (2016). Telomerase gene therapy rescues telomere length, bone marrow aplasia, and survival in mice with aplastic anemia. Blood.

[67] Jaijyan D.K., Selariu A., Cruz-Cosme R. (2022). New intranasal and injectable gene therapy for healthy life extension. Proc Natl Acad Sci U S A.

[68] Nagpal N., Wang J., Zeng J. (2020). Small-molecule PAPD5 inhibitors restore telomerase activity in patient stem cells. Cell Stem Cell.

[69] Solomon S.D., Adams D., Kristen A. (2019). Effects of Patisiran, an RNA interference therapeutic, on cardiac parameters in patients with hereditary transthyretin-mediated amyloidosis. Circulation.

[70] Bush A., Cunningham S., de Blic J. (2015). European protocols for the diagnosis and initial treatment of interstitial lung disease in children. Thorax.

[71] Kurland G., Deterding R.R., Hagood J.S. (2013). An official American Thoracic Society clinical practice guideline: classification, evaluation, and management of childhood interstitial lung disease in infancy. Am J Respir Crit Care Med.

[72] Deterding R., Young L.R., DeBoer E.M. (2023). Nintedanib in children and adolescents with fibrosing interstitial lung diseases. Eur Respir J.

[73] Cooney A.L., Wambach J.A., Sinn P.L., McCray P.B. (2021). Gene therapy potential for genetic disorders of surfactant dysfunction. Front Genome.

[74] Kormann M.S., Hasenpusch G., Aneja M.K. (2011). Expression of therapeutic proteins after delivery of chemically modified mRNA in mice. Nat Biotechnol.

[75] Kang M.H., van Lieshout L.P., Xu L. (2020). A lung tropic AAV vector improves survival in a mouse model of surfactant B deficiency. Nat Commun.

[76] Munis A.M., Hyde S.C., Gill D.R. (2021). A human surfactant B deficiency air-liquid interface cell culture model suitable for gene therapy applications. Mol Ther Methods Clin Dev.

[77] Munis A.M., Wright B., Jackson F. (2022). RNA-seq analysis of the human surfactant air-liquid interface culture reveals alveolar type II cell-like transcriptome. Mol Ther Methods Clin Dev.

[78] Chaytow H., Faller K.M.E., Huang Y.T., Gillingwater T.H. (2021). Spinal muscular atrophy: from approved therapies to future therapeutic targets for personalized medicine. Cell Rep Med.

[79] Angus D.C., Berry S., Lewis R.J. (2020). The REMAP-CAP (randomized embedded multifactorial adaptive platform for community-acquired pneumonia) study. Rationale and design. Ann Am Thorac Soc.

[80] Newton C.A., Zhang D., Oldham J.M. (2019). Telomere length and use of immunosuppressive medications in idiopathic pulmonary fibrosis. Am J Respir Crit Care Med.

[81] Kang C. (2019). Senolytics and senostatics: a two-pronged approach to target cellular senescence for delaying aging and age-related diseases. Mol Cells.

[82] Nambiar A., Kellogg D., Justice J. (2023). Senolytics dasatinib and quercetin in idiopathic pulmonary fibrosis: results of a phase I, single-blind, single-center, randomized, placebo-controlled pilot trial on feasibility and tolerability. eBioMedicine.

[83] Kinting S., Li Y., Forstner M., Delhommel F., Sattler M., Griese M. (2019). Potentiation of ABCA3 lipid transport function by ivacaftor and genistein. J Cell Mol Med.

